# Soldiers and marksmen under fire: monitoring performance with neural correlates of small arms fire localization

**DOI:** 10.3389/fnhum.2013.00067

**Published:** 2013-03-18

**Authors:** Jason Sherwin, Jeremy Gaston

**Affiliations:** ^1^Department of Biomedical Engineering, Columbia UniversityNew York, NY, USA; ^2^Human Research and Engineering Directorate, US Army Research LaboratoryAberdeen, MD, USA

**Keywords:** EEG, auditory oddball, expertise, military medicine, military personnel, generalized linear models (GLMs), source localization

## Abstract

Important decisions in the heat of battle occur rapidly and a key aptitude of a good combat soldier is the ability to determine whether he is under fire. This rapid decision requires the soldier to make a judgment in a fraction of a second, based on a barrage of multisensory cues coming from multiple modalities. The present study uses an oddball paradigm to examine listener ability to differentiate shooter locations from audio recordings of small arms fire. More importantly, we address the neural correlates involved in this rapid decision process by employing single-trial analysis of electroencephalography (EEG). In particular, we examine small arms expert listeners as they differentiate the sounds of small arms firing events recorded at different observer positions relative to a shooter. Using signal detection theory, we find clear neural signatures related to shooter firing angle by identifying the times of neural discrimination on a trial-to-trial basis. Similar to previous results in oddball experiments, we find common windows relative to the response and the stimulus when neural activity discriminates between target stimuli (forward fire: observer 0° to firing angle) vs. standards (off-axis fire: observer 90° to firing angle). We also find, using windows of maximum discrimination, that auditory target vs. standard discrimination yields neural sources in Brodmann Area 19 (BA 19), i.e., in the visual cortex. In summary, we show that single-trial analysis of EEG yields informative scalp distributions and source current localization of discriminating activity when the small arms experts discriminate between forward and off-axis fire observer positions. Furthermore, this perceptual decision implicates brain regions involved in visual processing, even though the task is purely auditory. Finally, we utilize these techniques to quantify the level of expertise in these subjects for the chosen task, having implications for human performance monitoring in combat.

## Introduction

In hostile environments where small arms fire is present, soldiers, and other operators (e.g., policemen), must rely on a rapid decision-making process that tracks the origin of a weapons fire, determine whether they are the intended targets, and finally decide how to respond. While automatic systems have been developed in recent years to assist in the localization of small arms fire (Bedard, 2006; Völgyeshi et al., 2007), these types of systems cannot fully replace the abilities of an unaided observer who must make rapid perceptual decisions in hostile environments.

Despite the practical importance of such observers to make decisions about small arms fire events, there has been little research addressing listener perception in this context. To the extent that this environment has been studied rigorously, perceptual analyses have been mostly limited to high-level metrics available from group dynamics measurements (Wilson et al., 2007) and descriptive surveys of friendly fire anecdotes (Hawley, 2008). The key point from the Wilson et al. study is that fratricide (i.e., friendly fire incidents) can be avoided when shared cognitive load is high, even when automatic localizers are used. There was no attention given to the actual neural processes underlying the perception of such an environment. In Hawley, there was even less consideration for cognitive factors.

To date, quantitative perceptual research on small arms firing events has focused on measuring and predicting hearing hazard from these intense impulsive events (Coles et al., 1968; Ward, 1968; Price, 2007). Closest to our aim in this study, Fluitt and Gaston have investigated listener ability to recognize and identify differences between small arms weapons for single-shot events (Fluitt, 2010; Gaston, 2012). There is at least one study that investigated listener ability to accurately localize a shooter across a limited range of conditions (Garinther and Moreland, 1966). Finally, more recently, Talcott and colleagues (Talcott, 2012) measured listener ability to localize a shooter based on the sounds of blank rounds, with and without the use of various hearing protectors.

Given the limited behavioral literature for the perception of small arms firing events, it is not surprising that to our knowledge, there is no work addressing the neural correlates of small arms fire events. The present work seeks to address this gap by measuring the neural correlates of subjects performing a listening task to differentiate the location of the sounds of recorded small arms fire. To this end, we modified the auditory oddball paradigm (Strobel et al., 2008; Goldman et al., 2009; Mangalathu-Arumana et al., 2012) where the target and oddball stimuli are firing events recorded from different relative locations. In a traditional auditory oddball paradigm, the difference between the targets and standards is obvious to a large population of subjects, but this is not necessarily the case for localizing arms fire, at least without possibly prolonged periods of preliminary training. This is due to the fact that in the absence of unambiguous spatial cues, the listening decision must be based on relatively subtle spectral differences created by different listening positions. This process of auditory source localization differs from earlier studies on the topic (not using small arms fire recordings) in which spatial location is communicated via inter-aural timing differences (Zatorre et al., 1999; Alain et al., 2001). In the absence of communicating location via these timing differences, our subjects were therefore recruited based on their extensive small arms experience, so that they could more readily perform the task using the subtle spectral differences of the stimuli.

In this paper we employ a forced-choice decision-making task, in which subjects must choose between the sounds of small arms being fired at forward-fire and off-axis “listener” positions in a timed task. We utilize a multivariate classifier to project the neural data (measured via EEG) into a space that optimally separates trials into their predicted angle class (Parra et al., 2005; Conroy and Sajda, 2012). By not limiting our analysis to specific electrode sites, we can derive what regions of the subjects' scalp activity indicate localization specific to infrequent incoming fire. Furthermore, we utilize source reconstruction techniques (Pascual-Marqui et al., 1999; Pascual-Marqui, 2002) to identify the neural generators of the decision-making process when the subjects correctly respond to forward-fire sound events. Finally, we utilize statistical hypothesis testing to investigate the extent to which neural activity predicts task performance. We also use this technique to determine whether self-reported expertise is a reliable predictor for either behavioral and/or neural performance metrics.

## Materials and methods

### Subjects

Eleven subjects (*N* = 11) participated in the study (two female, mean age −34.6 ± 9.0 years). All subjects were recruited based on the criterion that they have extensive experience using small arms fire. All subjects had either served in the US Armed Forces or were US government employees. All subjects self-reported experience using a mean of 7 ± 1 weapons. Of the subjects, eight self-reported the highest level of expertise (4) with at least one weapon on a scale of 1–4. All subjects reported normal hearing and no history of neurological problems. Informed consent was obtained from all participants in accordance with the guidelines and approval of the US Army Research Laboratory Institutional Review Board.

### Sound characteristics of small arms fire: differentiating targets and standards

We chose to use recordings of small arms fire as the stimulus event in an auditory oddball paradigm because of the temporal and spectral changes arising from the physics of a firing event from different relative locations. The sounds of small arms fire are the result of two events: (1) an explosive release of the buildup of pressure that propels a bullet from the weapon's muzzle and (2) the wake-like disturbance of air as the bullet moves toward the target. The direct acoustic consequence is an intense muzzle blast that propagates roughly spherically from the weapon's muzzle and has total duration of approximately 3–5 ms (Maher, 2006). As is true of the majority of small arms infantry rifles, if the bullet is supersonic, passage of the bullet through the air produces a sonic boom that propagates outward from the traveling bullet. This acoustic component is called a ballistic crack. It has a characteristic N-wave shape (peak pressure extremes correspond to the bow and stern of the traveling bullet), and has an extremely brief rise time (1–2 μs) and brief total duration (200–300 μs). As opposed to the spherically propagating muzzle blast, the ballistic crack propagates in a cone shape behind the bullet and expands away from the target line.

**Figure 1:**
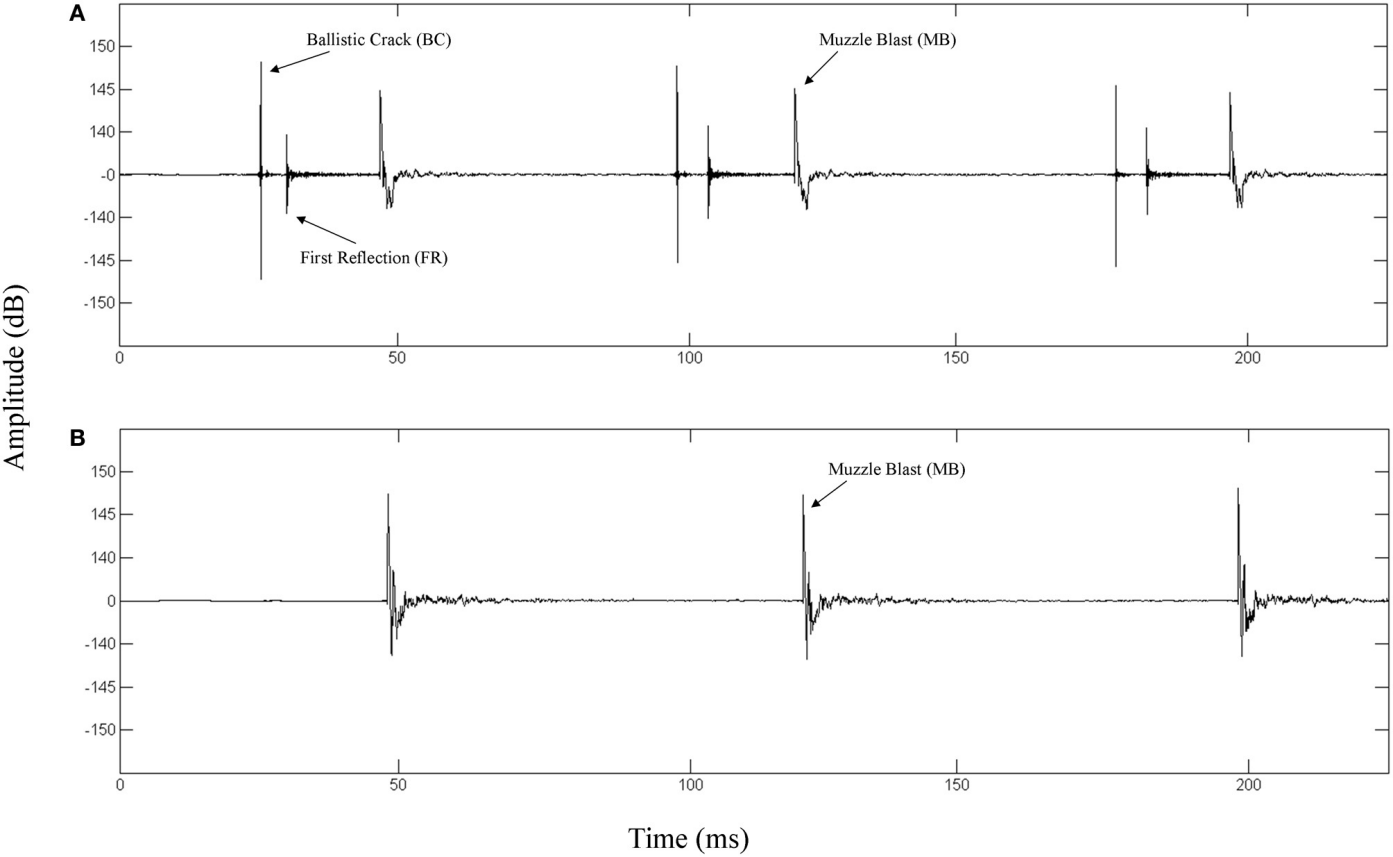
**(A)** Waveform of a 3-round burst of fire for a M4 carbine recorded 16 m in front of the shooter directly along the target line (0° incidence). The ballistic crack, ballistic crack reflection, and muzzle blast are labeled. **(B)** Waveform of a 3-round burst of fire for a M4 carbine recorded 16 m perpendicular to the left of the shooter target line (90° incidence). The muzzle blast is labeled.

The top panel of Figure 1 depicts the waveform of a fired M4 carbine measured at a position along the target line of fire. The bottom panel depicts the waveform of the same weapon being fired and measured at a position 90° to the left of the target line. The propagation of the ballistic crack occurs in a critical angle on either side of the target line, forward of the muzzle (Garinther and Moreland, 1966). Within the bounds of this critical angle, the ballistic crack is present and beyond this angle, the ballistic crack is absent. The critical angle is approximately 60° from the shooter to the left and the right of the target line. Based on these physical relationships, we can define two distinct listener positions: (1) A forward-fire position, where there is a ballistic crack followed by a muzzle blast, and (2) An off-axis position, where there is only a muzzle blast. Functionally, these two gross distinctions between listening positions can be indicative of relative safety (i.e., either nearer to, or further from, the shooter's target line of fire).

### Stimuli overview and behavioral paradigm

The basic audio stimulus set consisted of the recorded sounds (24 bit, 96 kHz) of an M4 carbine being fired at a small arms research facility at the US Army Aberdeen Proving Ground. Recordings were made at microphone locations 16 m directly in front of the shooter (0° relative to the shooter target line) and 16 m perpendicular to the shooter (90° relative to the shooter target line). Four 3-round bursts of fire were recorded at both of the microphone positions simultaneously. For the 0° stimuli, the average peak level of the ballistic crack was 149.4 ± 0.3 dB, and the average time between ballistic cracks and muzzle blasts was 20.3 ± 0.3 ms. Across both the 0° and 90° stimuli the average peak level of the muzzle blast was 149.3 ± 0.3 dB and the average time between muzzle blasts was 75.0 ± 0.5 ms. In addition to the firing events, three 120 s long recordings were made of the ambient background at the small arms range and the average level across the background was 71.0 ± 0.2 dBA. The actual audio levels presented to listeners were much quieter than these measured levels due to power constraints of playback speakers. Also, the playback level was such that the continuous noise levels never exceeded 85 dB and peak levels never exceeded 110 dB. Importantly for target detection, the average peak level of the firing sounds was greater than 78 dB above the average background noise level, and thus there was a signal to noise ratio of greater than 2:1 in each trial.

All stimuli were down-sampled to 16-bit, 44.1 kHz for experimental playback to subjects. Using a digital audio workstation (Logic Express 9.0, Cupertino, CA), audio scenes were created by mixing the eight unique firing events and three unique backgrounds. Each audio scene had at minimum 21 and at maximum 26 small arms 3-round bursts of fire. Two categories of firing events were used in each block, shots from 0° (targets) and 90° (standards). A sample audio scene is included as Supplementary Material with a key identifying the sequence of 0° and 90° shots (Table A1). The 0° shots were made to be the targets to more directly test perceptual decision making within the context of incoming fire. The firing events occurred on a jittered inter-stimulus interval (ISI) of 3104 ± 43 ms. There were a total of 663 stimulus events (513 standards, 150 targets) across the 27 unique audio scenes presented to subjects. The number of standards preceding a target in a sequence was 3.4 ± 0.3, with the minimum being 0 (12 times) and the maximum being 9 (1 time). The presentation order of scenes to subjects was randomized and no scene was heard twice.

The subjects were instructed to identify the relative angle of the firing event as quickly as possible via a keyboard button response, where each angle choice was mapped to a unique button (“1” and “2”). All button responses were executed with the right hand index and middle finger, regardless of handedness. After an initial training phase where subjects acclimated to the audio environment by hearing examples, and after a short initial practice session in which they responded with the button response and received feedback, the 27 blocks began and EEG data were recorded. During the EEG recording, the subjects received no feedback on their performance.

**Figure 2:**
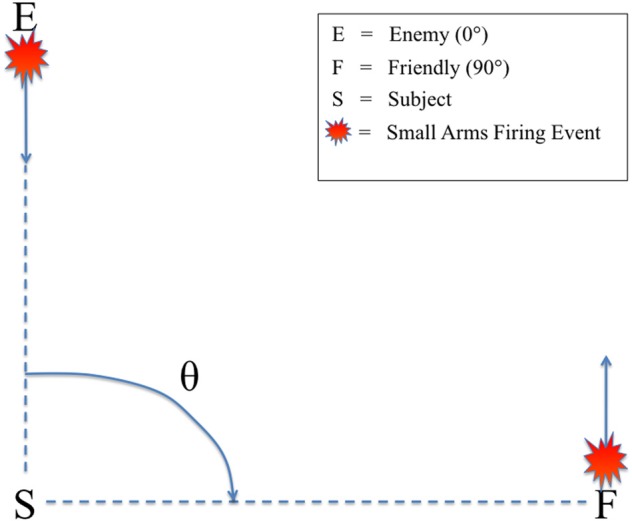
**Schematic of the simulated small arms fire localization task.** θ denotes the angle of incidence between the subject (S) and the firing event as conveyed by audio recording. Firing events from θ = 0° were labeled “enemy” (E) and θ = 90° were labeled “friendly” (F). Subjects were instructed to identify the angle of incidence of the firing event from these two possible choices as quickly as possible.

All audio stimuli were presented on a Dell A525 speaker system that included two satellite speakers placed to the left and right, directly in front of the subjects (12″ away), and a powered subwoofer placed on the floor in front of the listeners. There was no level panning to provide spatial information about the relative angle of the firing event. Rather, subjects could only determine the relative direction of the firing events based on non-spatial cues, specifically the presence or absence of the ballistic crack in the sound recording. Figure 2 shows the scene described to the subjects in which they were making decisions, with an enemy shooting up-range at an observer at a relative angle of 0° (forward-fire position) and a friendly shooting down-range from the observer at a relative angle of 90° (off-axis fire), both equidistant from the subject (16 m). Therefore, a button response to 0° was labeled “shoot back” and to 90° “all OK” to provide the subjects a more realistic experimental context. A Dell Precision 530 Workstation was used to present the audio stimuli with E-Prime 2.0 (Sharpsburg, PA). The subjects sat in an RF-shielded room with their eyes closed to minimize eye-blink artifacts. Despite increased alpha power, this technique has been used extensively in auditory perception tasks with EEG, mitigating any potential concerns of overpowering the ERP (Goldman et al., 2009; Maidhof et al., 2010).

The start of each 3-round burst of fire was the stimulus event by which EEG locking occurred. Stimulus events were passed to the EEG recording through a TTL pulse in the event channel. In *post-hoc* analysis, response events were added to the EEG via their latencies from the stimulus event.

### Data acquisition

EEG data were acquired in an electrostatically shielded room using a BioSemi Active Two AD Box ADC-12 (BioSemi, The Netherlands) amplifier from 64 active scalp electrodes. All channels were referenced to BioSemi's ground electrodes made for use with the Active Two. Data were sampled at 2048 Hz. A software-based 0.5 Hz high pass filter was used to remove DC drifts and 60 and 120 Hz (harmonic) notch filters were applied to minimize line noise artifacts. These filters were designed to be linear-phase to minimize delay distortions. Stimulus events—i.e., the first of the three-shot firing event—were recorded on separate channels.

In stimulus-locked epoching (−1000 to 1000 ms), the average pre-stimulus baseline was removed (−1000 to 0 ms). An alternative analysis also examined stimulus-locked epoching without an average baseline removal, but all results below are for the baseline-removed stimulus-locked epoching, unless otherwise indicated. After epoching to stimulus events, an automatic artifact epoch rejection algorithm from EEGLAB (Delorme and Makeig, 2004) was run to remove all epochs that exceeded a probability threshold of 5 standard deviations from the average. Similarly, in response-locked epoching (−1000 to 1000 ms), the average baseline was removed from −1000 to 0 ms and the same automatic artifact epoch rejection algorithm was run.

### Behavioral data analysis

We tracked behavioral performance in two ways, the first less stringent than the second. First, we tracked relative firing angle identification (i.e., forward fire, 0°, or off-axis, 90°) regardless of response time. Second, we enforced an additional criterion on angle identification that the response time had to be within 1000 ms of the stimulus onset. We chose this threshold because preceding analyses that employ overt responses and neural data discrimination do not consider trials that exceed this time after the stimulus onset (Goldman et al., 2009; Ratcliff et al., 2009; Sajda et al., 2009). Furthermore, the need to respond quickly to the stimulus provides a better simulation to combat. Therefore, we did the threshold analysis to more directly compare behavioral and neural results both with each other and with other similar experiments, all of which use this response time criterion. Thus, responses after 1000 ms from the stimulus event were deemed incorrect.

As a further analysis, we employed a statistical hypothesis test (paired *t*-test) on the response times without the time-threshold to determine if there was an inherent bias in response timing to either the target or standard stimuli.

### Neural data analysis

We performed a single-trial analysis of the filtered, epoched and artifact-removed EEG to discriminate neural response based on correct localization. To do so, we considered only behaviorally correct firing event responses, where the user's response was within 1000 ms of the first shot, and trained the classifier to discriminate a 90° relative firing angle (standard correct or SC) from one at 0° (target correct or TC).

Logistic regression was used as a classifier to find an optimal projection for discriminating between TC and SC conditions over a specific temporal window (Parra et al., 2002, 2005). Specifically, we defined a training window starting at either a pre-stimulus or post-stimulus onset time τ, with a duration of δ, and used logistic regression to estimate a spatial weighting vector ${\vec{w}}_{\tau ,\delta }^{T}$ which maximally discriminates between EEG sensor array signals *X* for each class:

Equation 1: projection equation for component

$$\vec{y}={\vec{w}}_{\tau ,\delta }^{T}X$$

In Equation 1, *X* is an *N* × *T* matrix (*N* sensors and *T* time samples). The result is a “discriminating component” $\vec{y}$ that is specific to activity correlated with each condition, while minimizing activity correlated with both task conditions. The term “component” is used instead of “source” to make it clear that this is a projection of all activity correlated with the underlying source. For our experiment, the duration of the training window (δ) was 50 ms. The window onset time (τ) was varied across time in 25 ms steps for both stimulus-locked and response-locked epochs, covering (0, 1000) ms in the former and (−1000, 1000) ms in the latter. We used the re-weighted least squares algorithm to learn the optimal discriminating spatial weighting vector ${\vec{w}}_{\tau ,\delta }^{T}$ (Jordan and Jacobs, 1994).

After solving for optimal discriminating spatial vectors in each window, there are additional analyses that we used for insight into our data. In order to provide a functional neuroanatomical interpretation of the resultant discriminating activity, and due to the linearity of the model, we compute the electrical coupling coefficients as shown in Equation 2. This calculation is also called the “forward model.”

Equation 2: sensor projection onto discriminating component

$$\vec{a}=\frac{X\vec{y}}{\vec{y}•\vec{y}}$$

This equation describes the electrical coupling $\vec{a}$ of the discriminating component $\vec{y}$ that explains most of the activity *X*. Therefore, $\vec{a}$ allows a topological representation of how strongly each electrode discriminates for one condition vs. another.

To complement the forward model analysis, we used the training window of optimum discrimination in epoch-time to inform an ERP-based source localization analysis. Specifically, we used the classification results of behaviorally correct trials (i.e., TC and SC). This was done on a subject-specific basis. Then we selected the window at which the leave-one-out *A*_*z*_ value was maximum for that subject. Using this marker in time, we trial-averaged the sensor data across all epochs that were either TC or SC, creating a grand average ERP for each subject and for each firing event angle. For 11 subjects and two conditions, this resulted in a total of 22 ERPs for each condition. Using grand average ERP values from subject-specific optimum windows, we utilized a source localization algorithm (sLORETA) (Pascual-Marqui et al., 1999) to calculate the most likely cortical source distributions. We did a paired *t*-test for TC vs. SC source distributions and calculated the resulting *t*-distribution of the log of the *F*-ratio using 2000 permutations to establish significance levels (*p* < 0.01) for the null hypothesis (h_0_: no difference in activity between TC and SC).

We quantified the performance of the linear discriminator by the area under the receiver operator characteristic (ROC) curve, referred to here as *A*_*z*_ using a leave-one-out approach (Duda, 2001). We used the ROC *A*_*z*_ metric to characterize the discrimination performance while sliding our training window from 0 ms pre-stimulus to 1000 ms post-stimulus (i.e., varying τ) for stimulus-locked and −1000 ms pre-response to 1000 ms post-response for response-locked. For stimulus-locked analysis, the former time period provided substantial time after the stimulus to observe any electrophysiological response to the firing event. For response-locked analysis, the latter time period provided ample time both before and after the behavioral response (button press) to observe any electrophysiological activity related to the decision in reaction to the firing event.

We quantified the statistical significance of *A*_*z*_ in each window (τ) by a relabeling procedure. Specifically, we randomized the truth labels between epochs of each class and retrained the classifier. For response-locked analysis, this was done 50 times for each of the 79 windows of each subject (*N* = 11), giving a total of 43450 permutations for a group level analysis. This number of permutations provides a large enough distribution to obtain a suitable number of samples after applying the Bonferroni threshold correction. Specifically, the *A*_*z*_ values from these permutations were used to establish a threshold for the *p* < 0.01/79 significance threshold. All significant results are thus reported at *p* < 0.01 Bonferroni corrected for multiple comparisons.

For stimulus-locked analysis, 250 permutations were done for each of the 39 windows of each subject. The false discovery rate (FDR) was then used within each window of each subject's epoch to adjust the *p* = 0.05 threshold line (Benjamini and Hochberg, 1995). The mean of this line across time points was then used as the corrected *p* = 0.05 significance line across the entire epoch within each subject. For group level analysis, we used the same procedure as was used for response-locked analysis, except subjects not discriminating after FDR correction were excluded from that analysis. Still, due to the higher number of within-subject label permutations (i.e., 250 stimulus-locked compared to 50 response-locked), we had a suitable number of permutations at our disposal for establishing the group-level Bonferroni thresholds.

### Combined behavioral and neural data analysis

We also investigated whether the neural data could predict the behavioral data. To this end, we used the Pearson correlation coefficient to determine a relationship between the within-subject number of FDR-significant discriminating windows and each behavioral metric reported in Table 1. We did this using data from all subjects.

**Table 1:** **Behavioral results and stimulus-locked discrimination summary**.

	**Subjects**										
	**S1**	**S2**	**S3**	**S4**	**S5**	**S6**	**S7**	**S8**	**S9**	**S10**	**S11**
Fraction TC	0.844	0.887	0.927	0.933	0.973	0.860	0.987	0.893	0.920	0.980	0.973
Fraction SC	0.790	0.988	0.612	0.994	0.994	0.998	0.998	0.990	0.994	0.996	0.998
Fraction TC (RT ≤ 1000 ms)	0.585	0.847	0.793	0.933	0.953	0.660	0.973	0.867	0.900	0.980	0.973
Fraction SC (RT ≤ 1000 ms)	0.594	0.938	0.497	0.988	0.951	0.848	0.944	0.959	0.990	0.977	0.986
Max. Az	N/A	0.69	N/A	0.73	0.68	N/A	0.67	0.71	0.72	0.72	0.76
Max. Az time (ms)	N/A	600	N/A	550	675	N/A	700	500	550	700	600
FDR significant windows	0	16	0	30	21	0	14	30	32	33	34

N/A, No FDR-corrected significant windows.

We also used the Pearson correlation to test the relationship between self-reported expertise and both neural and behavioral metrics of performance. Specifically, we correlated both a subject's number of reported weapons experience (mean 7 ± 1 weapons across our population) and his/her mean weapon experience (reported on a scale of 1–4, see “Materials and Methods”) with both neural and behavioral metrics of target discrimination. For our neural metric, we used the number of significant windows among those subjects showing stimulus-locked discrimination. For our behavioral metric, we utilized both TC and SC accuracy both with and without the time threshold.

## Results

### Behavioral performance

**Figure 3:**
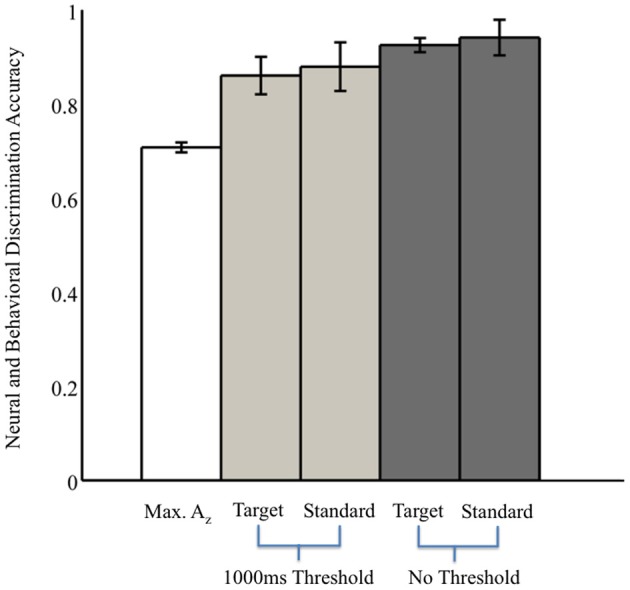
**Neural and behavioral discrimination accuracy across all subjects.** *A*_*z*_ values are reported from stimulus-locked epochs for all subjects with significant discrimination (false-discovery rate corrected, *p* < 0.01). Behavioral accuracy is also shown for all trials across all subjects, as well as response times within the 1000 ms threshold used for neural discrimination.

Without the threshold for response time, the right two bars of Figure 3 show that overall accuracy by event type was 0.93 ± 0.02 for 0° events and 0.94 ± 0.04 for 90° events. Though not shown, total accuracy regardless of event type was 0.93 ± 0.04. Requiring responses to be within 1000 ms of the stimulus, the behavioral data summarized in the light gray bars of Figure 3 show accuracy was 0.86 ± 0.04 for 0° events, and 0.88 ± 0.05 for 90° events. Even with the criterion on response timing, 8 of the 11 subjects had >94% of responses within this threshold (across all subjects, 92 ± 3%). Full details for all subjects' accuracies with and without the threshold can be seen in Table 1.

We also examined reaction times regardless of response accuracy. We found no difference in the mean response times to targets, 0° firing events, and to standards, 90° firing events (two-sample *t*-test, *p* = 0.71). Specifically, target response times were 712 ms (SEM, 37 ms), whereas standard responses were 689 ms (SEM, 39 ms), after the stimulus onset.

### Neural markers of correctly identified firing localization: stimulus-locked analysis

Table 1 shows the maximum *A*_*z*_ values and the number of FDR corrected significant windows for each subject. Time to maximum *A*_*z*_ was 609 ± 27 ms across subjects with significant discrimination. Of these subjects, the number of significant discriminating windows was 23 ± 3 out of a possible thirty-nine 50-ms windows. All subjects, except S1, S3, and S6, exhibit windows of neural activity discriminating for the target stimulus. These three subjects exhibit the lowest behavioral accuracies under the response-time threshold (see Table 1). Furthermore, they all exhibit the lowest three accuracies in either TC or SC across the population when there is no such threshold.

In an alternative analysis in which the baseline was not removed, only S3 exhibited no FDR corrected discrimination. This subject had the lowest accuracy of any other when there was a 1000 ms response time threshold (0.497 for SC).

Having this range of behavioral performance, we tested the relationship between neural discrimination and behavioral performance. We found positive correlation between number of significant windows and time-thresholded behavioral accuracy for 0° (*r* = 0.795, *p* < 0.01) and 90° (*r* = 0.812, *p* < 0.01) stimuli. The correlation disappears or weakens though without the response time threshold of 1000 ms: *r* = 0.545, *p* > 0.05 for 0° and *r* = 0.637, *p* < 0.05 for 90° stimuli. This result indicates the importance of the threshold as a means to concentrate neural activity in common time windows across trials so that discriminating activity can be found with the classifier. Importantly for performance monitoring in time-pressured situations, these results also indicate that neural activity can be used as a predictive indicator of behavioral performance.

We also investigated the extent to which self-reported expertise predicted neural and/or behavioral performance. The tested relationships can be found above in the Methods section, but for none of these single hypotheses did we find a significant correlation (*p* > 0.05 for each of them). Therefore, while we find that neural metrics can predict behavioral performance, we find no relationship between self-reported expertises as quantified in this study with either behavioral or neural performance.

**Figure 4:**
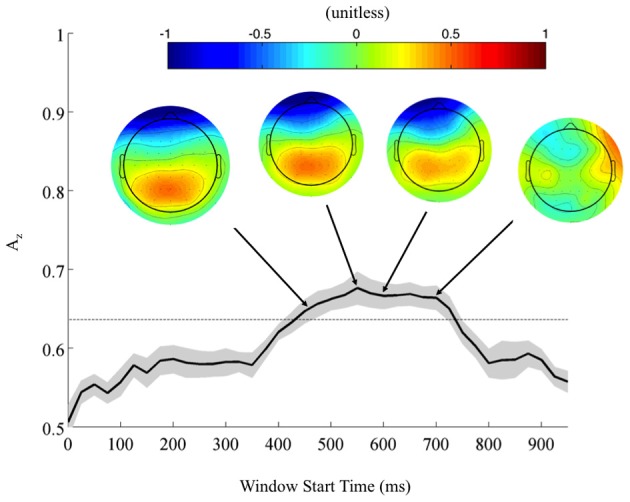
**Mean (black) and standard error (gray shading) stimulus-locked leave-one-out discrimination performance across all discriminating subjects (*p* < 0.01, Bonferroni corrected).** Included subjects had at least one false discovery rate (FDR) corrected window on a subject-level analysis. This leaves eight subjects from the original eleven, yet due to this criterion the discriminating window across subjects now more closely resembles the timing for the P300. Normalized forward models across discriminating subjects show the progression of significant discriminating activity, with higher values indicating discrimination for the targets (TC) and lower values for the standards (SC).

Returning to the FDR corrected discriminating subjects, we also found a common window of activity. By utilizing the 9750 permutations within each subject (hence, 78,000 across the discriminating eight subjects), we confirm the significance of this window at a *p* = 0.01, Bonferroni corrected threshold on a group level. Figure 4 shows this group of windows across the discriminating eight subjects, lasting from 450 to 725 ms. This result and the timing of peak *A*_*z*_ across discriminating subjects rests firmly in the latter portion of the P300 window (Linden, 2005). Accompanying forward model scalp projections confirm P300 activity in most of this time period.

From these results, we can also consider the possibility of a motor confound. Comparing the timing of this window (450–725 ms) to the response times summarized in Figure 3 (712 ± 37 ms, targets; 689 ± 39 ms, standards), we can see that the discriminating neural activity generally precedes the decision response. Consequently, it is likely that the discriminating activity is pre-motor and therefore, not due to a motor confound but rather, due to the subjects' ability to discriminate the sound characteristics of the different stimuli. Furthermore, forward model scalp topographies do not indicate sensors over motor areas in Figure 4.

Finally, from the perspective of performance monitoring, we find a lack of aural expertise in both the behavioral and neural response of three subjects from this analysis (S1, S3, and S6), despite these subjects' self-reported experience with small arms.

### Neural markers of correctly identified firing localization: response-locked analysis

**Figure 5:**
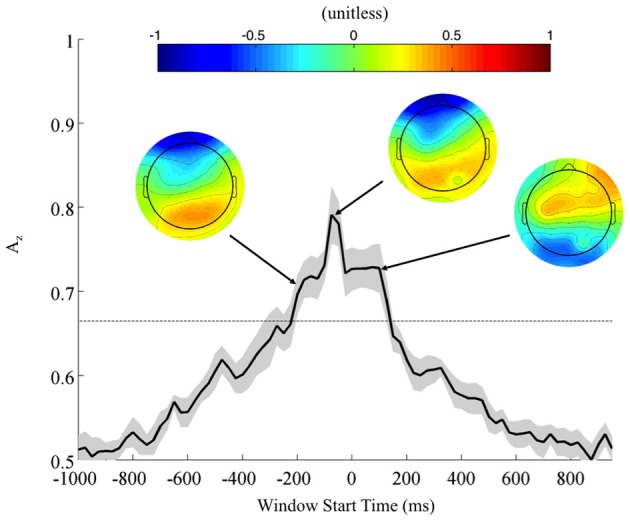
**Mean (black) and standard error (gray shading) response-locked leave-one-out discrimination performance across all subjects.** As opposed to the stimulus-locked leave-one-out analysis across subjects, we find a response-locked significant common window leading up to the decision and tapering off after it has been made (Bonferroni corrected, *p* < 0.01). Normalized forward models across subjects at indicated times show the progression of discriminating scalp activity, with higher values indicating target discrimination and lower values indicating standard discrimination.

To further investigate the possibility of a motor confound in the neural signal, we also classified EEG data locked to the response times (Figure 5). Once again, using correctly identified stimuli, we calculated the *A*_*z*_ values across all subjects. Accompanying forward models showing topological plots of target- and control-discriminating electrodes show that a right-lateralized P300 activity precedes the response and then yields to motor-related activity in the target condition. So while there is a possible motor component to the discrimination, it largely happens after the response and therefore cannot solely drive the discrimination of any perceptual decision-making preceding it.

In contrast to the stimulus-locked results, we find significant peaks (Bonferroni corrected, *p* = 0.01) across all subjects, including those exhibiting no stimulus-locked discrimination. For instance, all subjects (S1, S3, and S6 included) exhibit windows of highly discriminating activity leading up to or just following the response (maximum values of *A*_*z*_ for S1, S3, and S6 were 0.70, 0.79, and 0.96, respectively). So while these subjects can perform the task, the stimulus-locked results show that, in addition to exhibiting lower behavioral performance, they cannot do it with the same rapidity as the other eight.

In the context of the stimulus-locked group analysis, this response-locked group analysis demonstrates an evidence gathering process that proceeds at different rates for different subjects before executing their decision. By examining this process with response-locked epoching, we find that the decision process exhibits increasingly discriminating activity leading up to the response (peak at −75 ms, *A*_*z*_ = 0.79 ± 0.04), before tapering off as the response is executed. For subjects excluded from the stimulus-locked analysis, this decision process is more scattered in stimulus-locked time and therefore not observed. But its presence in response-locked time indicates that despite slow response times these slow-performing subjects exhibit discriminating neural activity when they do correctly localize. Furthermore, such slow-performing subjects had target accuracies greater than 84% when there was no response time threshold (see Fraction TC in Table 1 for these subjects), corroborating the claim that given enough time they could sufficiently perform the task, albeit at slightly lower behavioral accuracies than the other eight subjects. Nevertheless, the trial-to-trial temporal variability of these subjects' neural processes makes it difficult to discriminate TC from SC with stimulus locking.

### Forward models of discriminating activity

**Figure 6:**
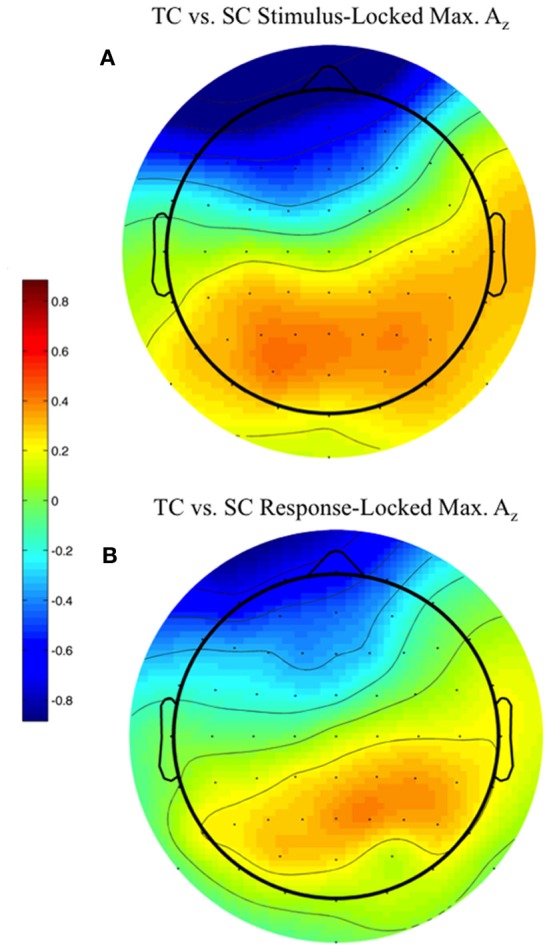
**Stimulus-locked (A) and response-locked (B) forward model across discriminating subjects at window of maximum A_*z*_ for front response-correct (target correct or TC) vs. side response-correct (standard correct or SC) discrimination.** Subjects are filtered by within-subject false discovery rate threshold (All subjects used in response-locked; S1, S3, and S6 removed from stimulus-locked). Forward model values (unitless) are within-subject normalized at each electrode site before averaging. More positive values indicate discriminating activity coupled with correct identification of the target stimulus, while more negative values indicate correct identification of the standard. The distribution of electrode coupling with target stimuli reflects in both locking conditions earlier work on auditory oddball scalp maps.

We used the maximally discriminating component activity to examine the spatial distribution of the neural response. Specifically, we estimated the electrical coupling $\vec{a}$ (i.e., the forward model) for each subject using behaviorally correct trials. Figure 6 shows these forward models averaged across all discriminating subjects (eight subjects for stimulus-locked in Figure 6A, 11 for response-locked in Figure 6B) at the window of maximum *A*_*z*_ (e.g., the times in Table 1 represent the stimulus-locked τ's for estimating the components in Equation 1 and the resulting forward models $\vec{a}$ using Equation 2).

We found spatial distributions showing a strong occipito-parietal component for the target stimuli (positive values = red, dimensionless) for both stimulus- and response-locked windows of maximum *A*_*z*_. Normalized by the within subject maximum value of $\vec{a}$, the forward model averages shown in Figure 6 are not dominated by one or an otherwise subset of the subjects.

### Source localization of discriminating activity

**Figure 7:**
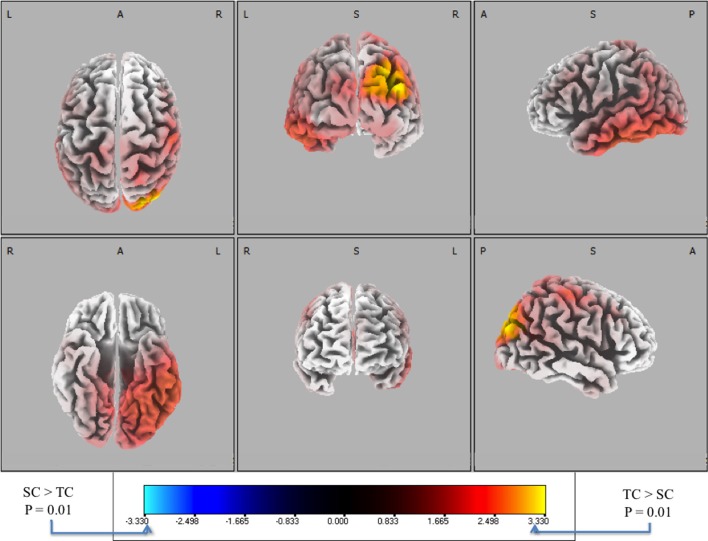
**Six-views of neuronal current paired *t*-tests (side response-correct (standard correct or SC) in purple/blue, front response-correct (target correct or TC) in orange/yellow) across all discriminating subjects.** In both plots, the t-distribution of the log of the *F*-ratio for each voxel is shown [*F*_(1, 20)_] with brighter colors indicating higher values of the *t*-statistic according to the color scale. Significance was established with a permutation test (2000 permutations). The EEG data used for these neuronal source calculations were the result of averaging scalp potentials at each channel at the subject-specific peak discrimination time. The correctly identified oddball stimulus (TC) is the only common activation across the subject population and the occipital activation in visual cortical areas resonates with previous findings in auditory oddball experiments (see “Discussion”).

To complement the forward model analysis, we used source localization to investigate the differences between behaviorally correct trials (i.e., TC vs. SC). Stimulus-locked data was not used because with only eight discriminating subjects there was not enough statistical power across the group to establish a significance threshold with statistical non-parametric mapping (Holmes et al., 1996). Therefore, response-locked data was used for all subjects because group-level analysis showed significant discrimination at multiple time windows (see Figure 5). Following the procedure described in the “Materials and Methods,” we utilized the sLoreta source localization algorithm and performed a paired *t*-test [*F*_(1, 20)_] to determine activity specific to TC or SC. We show the response-locked results from this hypothesis test in Figure 7.

As a check, we also ran a stimulus-locked analysis using classification from the non-baseline removed EEG data, since that analysis showed FDR corrected significant (though poorer) discrimination across 10 of the 11 subjects. Due to there being 10 subjects, we could establish a *p* < 0.01 significance line from permutation testing and statistical non-parametric mapping (Holmes et al., 1996). We found that the stimulus-locked distribution looked similar to Figure 7, though not as statistically significant (minimum voxel *p*-value was *p* = 0.012).

We used signed, one-tailed comparisons to evaluate the results against the null hypothesis (h_0_). Though the hypothesis of SC > TC shows no significant similarities (blue), a one-tailed *t*-test for TC > SC (red) shows a common neuronal current source located in the right visual cortex, particularly showing peaks in Brodmann Areas 19 and 39 (BA 19 and 39). In particular, the source activity peaks at MNI (−35, 80, 25), in the BA 19. While surprising that an auditory task would show common activity in the visual areas, there is precedent for it in fMRI bold activation (Goldman et al., 2009) and other auditory spatial localization studies (Alain et al., 2001). Finally, this result indicates right-lateralized common neuronal activity when subjects responded to the infrequent 0° relative angle firing event, i.e., when they “shoot back”. Since all subjects used their right index and middle finger to respond, this activity is not consistent with a motor response. Furthermore, since all subjects were utilized in the response-locked analysis, this result is invariant to the level of expertise as reflected by behavioral accuracies or response timeliness. Rather, the data indicate that this result is consistent with a common neuronal source when subjects aurally perceive infrequent incoming fire, an ability that may require some level of aural expertise, though not rapidity in decision-making.

## Discussion

In this paper, we have shown that there are underlying neural discriminators for localizing different infrequent firing events based on aural, non-spatial stimuli. However, our results diverge slightly from conventional auditory oddball results, as our experiment does. Specifically, we find that the timing of stimulus-locked neural discrimination falls in the latter portion of the expected timing window for a conventional oddball task, whereas the response-locked trends are similar to those found in conventional stimuli experiments. When focusing the analysis on subject-specific neural discrimination, we found that scalp activity discriminates for the target stimuli much like what is seen in auditory oddball tasks. We also showed that there appears to be a common neural generator when subjects correctly responded to the infrequent 0° forward-fire stimuli. Finally, we find a relationship between behavioral and neural indicators of performance, having implications for human performance monitoring and training in time-pressured environments (e.g., combat). We will now review these results in the context of the relevant literature.

### A neural marker for infrequent firing event localization

To our knowledge, there have been no results published on the neural markers of small arms fire localization. There is however, an available literature on automatic, i.e., non-human, systems for localization (Bedard, 2006; Völgyeshi et al., 2007). These systems utilize assumptions about the physics of sound propagation due to a firing event (e.g., muzzle blast and ballistic crack) to determine the most likely point in three-dimensional space from where the shot originated. Here, we have limited the task to localization in two dimensions (i.e., there is no azimuth). Also, we have specifically considered subjects with experience in arms fire due to the level of aural expertise likely necessary to distinguish between the stimuli categories, especially in the absence of spatially localized sound sources. The goal in our analysis has not been to make a more perfect localizer; rather it is to determine what neural activity, if any, occurs when such localization occurs in an environment simulating the situational awareness of soldiers in a hostile theater.

There has been some work on the perception of small arms firing events at different firing angles (Fluitt, 2010; Gaston, 2012). But the focus of that work was on discrimination and identification of the weapon type fired, rather than weapon localization. No neural data were recorded either. In the few instances where small arms localization was studied, the events were either produced by blanks which are impoverished analogs to real gunfire (Talcott, 2012) or localization was assessed under limited conditions (Garinther and Moreland, 1966). Once again, no neural data were recorded in these studies. Consequently, it is difficult to validate our findings in the context of this and other parallel research.

Another area of study that does provide a reference point is auditory oddball research, especially because of our choice for experimental paradigm. In particular, Goldman et al. investigated the neural correlates of auditory oddball tasks with simultaneous EEG and fMRI (Goldman et al., 2009). In this study, and other auditory oddball ones (Strobel et al., 2008; Goldman et al., 2009; Mangalathu-Arumana et al., 2012), there is a rare (target) stimulus that occurs with variable frequency amidst otherwise repeated common (standard) stimuli. In such tasks, the frequency characteristics of the stimuli are simple and all standards/targets are spectrally similar. This was not the case though for our experiment, in which standards are sampled from four unique recordings of an M4 carbine fired at a 90° relative angle (off-axis fire) from a distance of 16 m. Similarly, our targets were also sampled from four unique recordings at 0° (forward-fire) from a distance of 16 m. Finally, conventional auditory oddball tasks incorporate no background stimuli, as we have done by mixing three unique background stimuli with nine unique firing sequences to create 27 unique audio scenes.

Despite this added level of aural complexity, we find that most neural results we found have analogs in the conventional auditory oddball paradigm. For instance, Goldman et al. found stimulus- and response-locked forward models comparable to the ones we found (compare Figures 6A,B to Figures 6 and 7 in Goldman, for stimulus- and response-locked, respectively), exhibiting left-lateralized occipito-parietal activity in the former and right-lateralized in the latter. Similarly, a conventional auditory oddball task exhibits group-level common windows of discrimination for response-locked analysis (see Figure 2 in Goldman), as we found (Figure 5). We see similar trends for stimulus-locked analysis, as we found common windows of discrimination across those subjects that showed within-subject FDR corrected discrimination (Figure 4). However, these common windows fall in the latter portion of the P300 (Linden, 2005). For these subjects, accuracy results are closer to those observed in conventional oddball tasks (e.g., Goldman et al.).

Finally, the connection we find between behavioral performance and neural discrimination (via number of FDR significant windows) echoes previous work linking these complementary measures of evidence gathering processes (Ratcliff et al., 2009; Sajda et al., 2009). But our point of departure from these earlier studies is the self-reported high level of small arms expertise amongst our subjects. Nevertheless, despite some subjects' claims of expertise in small arms fire, we find that neural discrimination predicts the poor performance found in time-pressured response accuracy for these subjects (e.g., S1, S3, and S6). Furthermore, we found that self-reports of small arms experience are not a predictor of either neural or behavioral metrics of performance on this task.

### Spatial localization vs. oddball response

The use of the oddball paradigm both helps and hinders the formation of conclusions as to what extent the neural signal we have found is indicative of either arms fire localization or a purely oddball response. For instance, location is communicated via the stimuli's spectral characteristics, especially if a subject's auditory expertise in small arms fire draws on this association. Conversely, the oddball paradigm provides an orienting response in the subject when the infrequent stimulus (one of the four 0° shots) occurs, providing a clear neural response characterized by the P300. But we do not find a canonical P300 amongst our stimulus-locked discriminating subjects (Figure 4) in terms of timing. In particular, while the scalp topology is consistent with a P300 (Linden, 2005), the timing is delayed from that shown in other auditory oddball paradigms in which simpler stimuli were used (see earlier reference to Goldman et al.). Although beyond the scope of this current study, we hypothesize that this deviation from a classic oddball response is due to the implicit meaning carried by our stimuli to the expert subjects within our population. Specifically, for those experienced in small arms fire, not only do these stimuli carry implicit spatial information (as demonstrated in Figure 1), but also the simulated scenario in which they were presented lays a cost on misidentification. Even though feedback was not given after each trial to provoke a potential reward-punishment response, there is an implicit cost/benefit relationship in returning fire to friendly fire (90° stimuli) or not returning it to hostile fire (0°). Therefore, both the spatial information and the implicit valence of the stimuli could be more apparent to an expert population than to a novice one.

While a subsequent study would benefit from a corresponding novice population to settle this hypothesis' veracity, we can find insight into the extent to which our response is oddball, localization, or both from additional literature on spatial localization. For instance, Alain et al. (2001) show distinct “what” and “where” neural pathways from both EEG and fMRI studies on object recognition and spatial localization, respectively. We find similar activity from source localization (Figure 7) to the blood-oxygen-level-dependent (BOLD) signal they find (see Figure 2 of Alain et al.) in the visual areas for auditory spatial localization. But a primary difference in their paradigm from ours is important: they simulate spatial location of an auditory source via a head-related transfer function (HRTF) (Wenzel et al., 1993), whereas we communicate spatial location via the spectral nuance of the four 0° and four 90° stimuli. While the difference is subtle, it cannot be underestimated for its potential impact on neural circuitry involved in task performance.

The primary distinction between our study and traditional localization results lies in the type of evidence presented to the auditory system. In spatial localizations studies, the HRTF simulates the inter-aural delay caused by a sound source in three-dimensional space. This delay provides the bulk of the information by which localization occurs, regardless of the source's spectral features. But in our study, the stimuli from both 0° and 90° were always presented from stereo speakers in front of the subject, thereby removing the possibility of discrimination from inter-aural delay. Rather, the differences in timbre (illustrated in Figure 1) communicate localization to the trained ear.

In contrast to our paradigm, earlier studies on auditory localization have separated object recognition and spatial localization via stimuli and/or task. For instance, Alain et al. utilize different stimuli for object recognition (“what”) and spatial localization (“where”), thereby easily bifurcating the two neural circuits responsible for each in an fMRI analysis. Others who have investigated sound localization, such as Zatorre et al. (1999), have used the same stimuli but have alternated the task between object recognition and spatial localization to do similar bifurcation. However, in the terminology of Alain et al., we use auditory object recognition (the “what” pathway) to inform spatial localization (the “where” pathway). Of course, the connection between object recognition and spatial localization is only guaranteed if the subjects' experience provides the context to make it. As we used a small arms expert population (at least by self-report), it is possible that the source activations we find in visual cortices in response to correct identification of 0° stimuli are due, to some extent, to the “where” pathway, although the oddball response seems to have a dominating role. Once again, a definitive answer on this hypothesis would result from an expert vs. novice comparison, which is beyond the scope of this current study.

### Aural event discrimination in visual cortices

The other major finding of this paper is that on correctly identified firing events there is a common neuronal current source active across our population. The timing for this activity is determined from the peak discrimination within each subject; therefore it is controlled for the variability between subjects. The common neuronal activity is found both from the stimulus- and response-locked discrimination between TC and SC. But we have only shown the response-locked results due to there not being enough FDR corrected discriminating subjects in stimulus-locked epoching to provide a statistically rigorous hypothesis test. Even though the timings of the response-locked discrimination peaks are close to the response, we see no evidence in the common neural generator that the discriminating source is motor-related. Rather, the common neuronal current source, which is peaked at MNI (−35, 80, 25) in Brodmann Area 19 (BA 19), is unequivocally visual-spatial in its location (Jonides et al., 1993), and corroborates the forward model (Figure 6B).

There are two equally plausible, yet still unsubstantiated, explanations for this result. Goldman et al. propose that lateral occipital cortex (LOC) activation in an auditory task could occur due to modulations of attention across the brain when the subject orients to the target stimulus. An alternative explanation would be that there is implicit spatial information in the audio stimuli used in our experiment, causing the subjects to have common visual-spatial cortical activation in response to the aural cues used in our experiment. The earlier contextualization of our results in auditory spatial localization literature (Zatorre et al., 1999; Alain et al., 2001) adds support to this interpretation. Nevertheless, none of these explanations suffices without a precisely designed experiment that can remove the overt or even implicit mappings of auditory stimuli to spatial domains (e.g., mapping tones to a number line in Hz). This might be accomplished with a corresponding novice population for whom the stimuli are not likely to evoke implicit spatial cues.

### The potential role of small arms expertise

Our selection of a population with substantial self-reported small arms experience raises the question as to what extent this choice has driven our findings. In no way do we claim that these findings are exclusive to a population of subjects with such experience. Rather, in this initial study of the neural correlates of small arms localization, we have started with an expert population because of the potential difficulty in learning to distinguish the 0° from the 90° stimuli (see “Supplementary Material” for examples). For instance, although not formally reported here, pilot testing of the paradigm on non-experts revealed the potential need for extensive pre-experiment training to distinguish the stimuli categories. Therefore, to more expediently study our hypothesis—i.e., that neural response could predict behavioral response and provide a quantitative check on self-reported expertise—we sought an initial population for whom the learning curve would be minimal due to their prior experience with small arms sounds.

While we do find potential markers for expertise in the temporal delay of the P300 (see earlier in “Discussion”), and a gradation of performance at the task among our expert population, we cannot rule out the possibility that this task may be done with a high level of accuracy by a novice population. In fact, within our current study, we investigated the possible relationship between experience and performance by using the self-ratings on weapon expertise provided by the subjects and found no relationship (*p* > 0.05 for all Pearson correlation tests).

To address a possible concern that age effects could have steered behavioral and neural performance trends, we did a similar test against self-reported age, rather than either metric of expertise. We found no correlation (*p* > 0.05, Pearson correlation test) between age and either neural or behavioral performance.

Finally, while it is possible that members of our subject population have hearing damage built over years of exposure to arms fire, we do not suspect that this is the driving factor behind performance on a population level. 8 of 11 subjects exhibit stimulus-locked neural discrimination and all subjects do so on a group-level for response-locked analysis. This population-level and within-subject performance result indicates other factors than hearing ability (e.g., loss of attention) degrade performance in under-performing subjects.

## Conclusions

In summary we have identified neural markers that can be used to determine when (and if) subjects highly trained in the use of small arms fire classify the relative angle of audible shots with variable event frequency. We have identified the timing of this decision with respect to the stimulus onset (i.e., the first audition of the firing event) and to the motor response. We found that all subjects utilized an evidence-gathering process whose discriminating activity peaked when leading up to the decision. We also found that behaviorally fast performing subjects exhibited common windows of discrimination with respect to the stimulus, whereas slow performing subjects did not, implying that such neural discrimination can be a measure of expertise at the task. We further find that neural discrimination in these subjects predicts reduced behavioral performance and that self-reports of expertise are not a predictor of good performance.

We also identified BA19 as an activated region during accurate target trials, i.e., when incoming firing events were discriminated. Comparison to previous auditory oddball research shows that activation in this area is not unique to localizing small arms fire. Rather, it is a feature of the neural response to the oddball event. Still, the utility of detecting this response cannot be underestimated for combat situations, in which infrequent yet important orienting events (such as incoming fire) require accurate and rapid decision execution. Neverthless, future work will attempt to separate the extent to which the neural response found in this study is more due to the oddball or the implicit spatial information carried by the stimuli. We hypothesize without demonstrating here that an expert vs. novice study using the same paradigm and similar analytic techniques can provide an answer to this question, as the novice population will have had no prior experience linking the stimuli to spatial cues.

An embedded future research question generated by this work is how do experts compare to novices on a neural level? If they do differ, can we track the development of expertise? Even within our population of experts, we find a gradation of expertise for the task of arms fire localization from auditory cues and a set of both neural and behavioral metrics to check self-reported expertise. Additionally, an expert vs. novice study may even reveal a neural advantage among some novices for doing the task when such expertise had not otherwise been noticed. This knowledge can greatly facilitate soldier training for combat.

Finally, other future work will focus on the application of this technique to weapon identification and integrating other cues, such as visual. With this methodology, we will be able to show what role additional cues play in both localization and enemy/friendly identification. For instance, in our study, the valence and direction of the stimuli are conflated, as all 0° stimuli are labeled “enemy” and 90° stimuli are labeled “friendly,” both from the same weapon. Future work could place both enemy and friendly weapons (e.g., an AK and an M4) at 90° and 0°, thereby making the discrimination task more difficult, more informative for bifurcating “what” and “where” neural circuitry, and more useful for real-world combat application. This initial study has laid the groundwork for such a study, having introduced a host of signal detection and statistical hypothesis testing techniques that can be therein employed. Executing these future studies may give us insight into better performance monitoring and more efficient strategies in recognizing friend and foe on the battlefield.

### Conflict of interest statement

The authors declare that the research was conducted in the absence of any commercial or financial relationships that could be construed as a potential conflict of interest.

## Appendix

**Table A1:** **Event key for example audio scene**.

Event number	1	2	3	4	5	6
Angle (°)/Take number	90/3	0/1	90/4	90/1	90/1	0/3
Time (ms)	1749	4705	6546	9795	13,211	16,829
Event number	7	8	9	10	11	12
Angle (°)/Take number	0/3	90/2	90/2	90/2	90/2	0/3
Time (ms)	20,139	23,665	27,104	30,237	33,364	36,495
Event number	13	14	15	16	17	18
Angle (°)/Take number	90/2	0/2	90/2	90/2	90/3	0/3
Time (ms)	40,732	44,462	47,512	50,638	53,668	56,657
Event number	19	20	21	22	23	24
Angle (°)/Take number	90/4	90/4	0/1	90/2	90/2	90/4
Time (ms)	60,105	63,522	67,000	70,420	73,754	77,573

Event numbers and angles of small arms firing event are listed with one of four unique recording numbers. Time from the beginning of the recording is shown with each event.
